# An Integrated Preprocessing Approach for Exploring Single-Cell Gene Expression in Rare Cells

**DOI:** 10.1038/s41598-019-55831-2

**Published:** 2019-12-24

**Authors:** Junyi Shang, David Welch, Manuela Buonanno, Brian Ponnaiya, Guy Garty, Timothy Olsen, Sally A. Amundson, Qiao Lin

**Affiliations:** 1 Department of Mechanical Engineering, Columbia University, New York, New York, 10027 USA; 2 Center for Radiological Research, Columbia University Irving Medical Center, New York, New York, 10032 USA

**Keywords:** Biophysics, Biotechnology

## Abstract

Exploring the variability in gene expressions of rare cells at the single-cell level is critical for understanding mechanisms of differentiation in tissue function and development as well as for disease diagnostics and cancer treatment. Such studies, however, have been hindered by major difficulties in tracking the identity of individual cells. We present an approach that combines single-cell picking, lysing, reverse transcription and digital polymerase chain reaction to enable the isolation, tracking and gene expression analysis of rare cells. The approach utilizes a photocleavage bead-based microfluidic device to synthesize and deliver stable cDNA for downstream gene expression analysis, thereby allowing chip-based integration of multiple reactions and facilitating the minimization of sample loss or contamination. The utility of the approach was demonstrated with QuantStudio digital PCR by analyzing the radiation and bystander effect on individual IMR90 human lung fibroblasts. Expression levels of the Cyclin-dependent kinase inhibitor 1a (*CDKN1A*), Growth/differentiation factor 15 (*GDF15*), and Prostaglandin-endoperoxide synthase 2 (*PTGS2*) genes, previously shown to have different responses to direct and bystander irradiation, were measured across individual control, microbeam-irradiated or bystander IMR90 cells. In addition to the confirmation of accurate tracking of cell treatments through the system and efficient analysis of single-cell responses, the results enable comparison of activation levels of different genes and provide insight into signaling pathways within individual cells.

## Introduction

Rare cells, considered as low-abundance cells (<1000/mL)^1^, are highly important for many applications including disease diagnosis, cancer research and environmental analysis^2^. Rare cells of interest include circulating tumor cells (CTC), circulating fetal cells, and stem cells. For example, intact fetal cells in maternal blood can be isolated and characterized for noninvasive prenatal diagnosis^3^. Isolation and analysis of CTCs is critical for diagnosis and prognosis of many cancers as well as estimating the risk of metastatic relapse^4^. Single-cell analysis, which has been extensively used for examining cell heterogeneity, may serve as an important tool for detecting cell-to-cell variation or responses in rare cells. Cell heterogeneity, as manifested by different stages, genetic lesions, or expression programs, is associated with distinct outcomes or therapeutic responses. For example, cells from the same tumor may exhibit distinct phenotypic or epigenetic states, and such intratumoral heterogeneity can cause treatment failure and recurrence of disease^5^. Due to the increasing awareness of analyzing cellular response on a cell-by-cell basis^6–9^, methods for single-cell gene expression profiling, which assay gene patterns in individual cells, have been developed to assist in the study of rare cell expression variability and enable characterization of intracellular molecular mechanisms and pathways^10^.

Single-cell gene expression analysis has also proved useful for studying the cellular response to ionizing radiation^11^. Ionizing radiation is commonly used as a probe of cellular damage and repair mechanisms. Following ionizing radiation, cells activate biochemical pathways that consist of DNA damage cell-cycle checkpoint pathways and the DNA repair pathways, which promote cell survival while keeping DNA integrity^12^. Diverse cellular activities are engaged in the components of these pathways, such as apoptosis, cell cycle arrest, stress signaling and DNA repair^13^. These responses may be induced by alterations in protein modification or changes in subcellular localization as well as changes in gene expression profiles^14–16^. For their central role in elucidating the mechanisms underlying cellular radiation response, alterations in gene expression have been studied extensively in populations of cells post irradiation^17–19^. While few gene expression studies have covered the cellular response of individual cells to ionizing radiation, gene expression patterns in single irradiated cells have been explored via conventional quantitative RT-PCR^20^. A recent approach of using low-density Taqman real-time PCR has extended the ability of quantitative measurement from 3 genes to 48 genes in one single irradiated cell^11^. An interesting application of single-cell gene expression analysis in radiation studies is exploring the bystander effect, in which non-irradiated cells demonstrate several responses seen in nearby irradiated cells. While this appears to be mediated by signal transmission between irradiated and nearby non-irradiated cells via direct physical contact^21^ or through the culture medium^22^, the exact nature of the signal and the mechanisms of stress transmission remain to be determined. These bystander mechanisms have implications for the assessment of radiation risk in situations from low-dose environmental exposures, to particle therapy for cancer, to manned Space missions^23^. The microbeam facility at the Center for Radiological Research is particularly well suited for more definitive study of the bystander effect^24^. While a low dose of particle radiation delivered to a cell population follows a Poisson distribution^20^, allowing calculation of the percentage of cells not directly traversed by a particle, the microbeam can deliver a precise number of particles (including one) to a specified cell in the population. Therefore, the microbeam enables irradiation with a precise number of particles to each individual hit cell, while the non-hit single cells are not traversed by a particle.

Following the microbeam irradiation of single cells, microfluidic devices can be used to facilitate handling the preprocessing functions (e.g., cell lysis, RNA purification, etc.) for gene expression measurements. Microfluidic platforms featuring rapid and sensitive biochemical synthesis and analysis that also allow automation, integration and parallelization, have been increasingly used for gene expression profiling^25–27^. To date, microfluidics for single-cell gene expression have integrated functional components involving single cell isolation and lysis^28^, RNA purification^29^, and RT-PCR^30^. While demonstrating a modular and integrable platform, a highly integrated microfluidic tool requires complex device design and complicated off-chip control instrumentation. For example, microfluidic single-cell isolation that uses valve-controlled chambers^31^ relies on multilayer soft lithography, increasing the complexity of fabrication and pneumatic control. Microfluidic droplet-processing single-cell analysis devices typically require complex control with heating modules, particularly with the integration of droplet generation and droplet thermo cycling^32^. We have previously developed a bead-based approach for performing RT-qPCR at the single-cell level on a microfluidic device, which featured integrated cell trapping, cell lysis, RT and qPCR on chip with high sensitivity and efficiency of the RT-qPCR reaction compared to off-chip RT-qPCR^33,34^. While this approach has demonstrated sensitivity and efficiency in single-cell gene expression analysis, one limitation is that the cell trapping unit uses a geometric constraint to capture one single cell out of a group of cells, which does not allow tracking the identity of an individual cell. Commercial options also exist for performing single-cell gene expression analysis, such as the C1/BioMark system (Fluidigm, inc). Unfortunately, the input cells are sorted randomly, which hinders the tracking of individual cells from the time of irradiation to analysis.

This paper presents an approach that combines single-cell picking, lysing, reverse transcription (RT) and photocleavage to enable the isolation, tracking and gene expression analysis of rare cells. A single-cell capillary picker introduces single cells into a microfluidic device, termed the Preprocessing for Gene-Expression Measurement (PreGEM) chip, which performs gene-expression preprocessing. This approach permits the isolation and quick lysis of individual cells, while avoiding the complex microenvironment required for culturing cells on chip^35^, thereby enabling the study of microbeam-irradiated single cells and bystander effects, or other rare cells. The PreGEM chip enables synthesizing and delivering stable cDNA for downstream gene expression analysis. The chip tracks the identity of individual cells in a single microfluidic channel, which is readily scalable by parallelization. A bead-based protocol is used to achieve mRNA capture and the RT reaction, greatly simplifying both the design and operation of the microchip. Furthermore, instead of applying thermal denaturation (95 °C) to release single-stranded DNA (ssDNA), which tends to cause evaporation issues^36^, a photocleavage approach has been used to release cDNA from the beads realized via a photocleavable link between the bead and cDNA. Finally, the approach maximizes the choice of platforms for downstream analysis, such as qPCR real-time gene expression analysis, QuantStudio digital PCR sensitive detection or NanoString multi-gene expression analysis. We demonstrate the preprocessing approach using QuantStudio digital PCR by measuring expression levels of the radiation responsive genes^37,38^ Cyclin-dependent kinase inhibitor 1a (*CDKN1A*) and Growth/differentiation factor 15 (*GDF15*) as well as of the Prostaglandin-endoperoxide synthase 2 (*PTGS2*) gene (both radiation and bystander responsive^39^) in individual control, microbeam-irradiated or bystander IMR90 human lung fibroblasts after 4-hour co-culture. The results confirm the ability of the preprocessing approach to enable accurate tracking of single cells and efficient analysis of single-cell responses, as well as allow the comparison of activation levels of different genes and signaling pathways within individual cells.

## Results

We first performed on-chip RNA capture validation, then characterized on-chip RT efficiency and bead-based reaction efficiency, as well as quantifying the releasing efficiency of cDNA from beads. We then demonstrated single-cell analysis with the integrated system from microbeam irradiation, cell picking, and on-chip reactions, all the way through QuantStudio dPCR. The integration of the system allows the exploration of cell-to-cell variability in response to microbeam irradiation and in bystander cells.

### Validation of RNA capture on the Pre-GEM microchip

**Figure 1:**
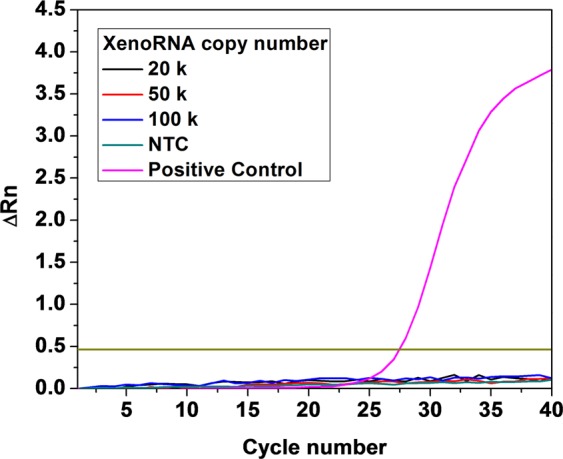
Quantified detection of RNA trapping efficiency using 3.5 × 10^6^ beads. There was no detectable residual XenoRNA template in the binding waste.

Before characterizing efficiencies of downstream reactions, the on-chip capture of mRNA via 5′-PC-Biotin-(dT)_25_-3′ Streptavidin magnetic beads was validated using XenoRNA template. The value of ΔR_n_, indicating the magnitude of the fluorescent signal and, therefore, amplification generated by PCR, was 3.7 for the positive control (10^5^ XenoRNA with 3.5 × 10^6^ beads) after 40 cycles of bead-based PCR. Referring to the method section, the remaining solutions after bead capture were mixed with bead-based solution, and RT-PCRs were performed to quantify remaining templates in the solution after the first capture. It was found that the ΔR_n_ values remained below the threshold (Fig. 1). Thus, we can conclude that 3.5 × 10^6^ beads were sufficient to capture essentially all of the polyAdenylated templates, and an undetectable amount of free RNA templates were residual in the binding waste.

### On-chip RT characterization and interference of beads with reaction efficiency

**Figure 2:**
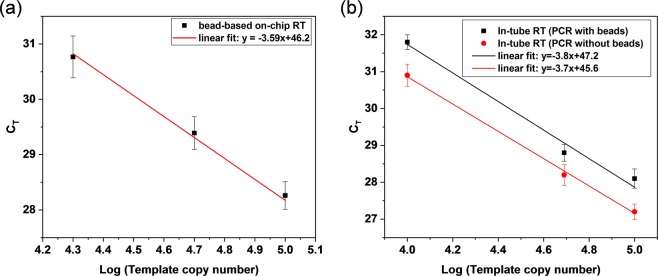
(**a**) Mean and standard deviation of on-chip bead-based RT followed by offchip bead-based qPCR. (**b**) Mean and standard deviation of in-tube RT followed by bead-based offchip qPCR and solution-based offchip qPCR, respectively.

Starting with 20k, 50k and 100k copies of XenoRNA, the on-chip bead-based RTs followed by off-chip bead-based qPCRs were conducted to test repeatability and characterize reaction efficiency (Fig. 2a). We found that under the given experimental conditions, the PCR efficiency defined by (10^−1/*k*^ −1) × 100%, where *k* is the slope of the *C*_*t*_ as a function of the logarithm of the template copy number, for our bead-based approach was 90.5%. To characterize the on-chip RT efficiency, we conducted the bead-based PCR testing following in-tube RT. The PCR efficiency *E*_b_ for the bead-based PCR with in-tube RT was 83.2%, meaning the bead-based on-chip RT has a 1.08-fold increase in efficiency compared with that of in-tube RT. This improved efficiency for on-chip reaction likely resulted from more efficient molecular interactions in the microscale environment^25,40^. Meanwhile, we ran a solution-based PCR without introducing beads following in-tube RT. Some bead interference was observed, affecting the reaction efficiency, as the presence of beads during the PCR reaction yielded higher *Ct* values than the solution-based reactions. In terms of the reaction efficiency, it was found in Fig. 2b that under the given experimental conditions, the PCR efficiency *E*_s_ for the solution-based PCR testing (86.3%) was higher than the PCR efficiency *E*_b_ for the bead-based PCR (83.2%), which was likely attributable to enhanced molecular interactions in the absence of the beads. The reaction efficiency was not dependent on template copy number, however. Based on these results, we calculated a correction factor that can be used to estimate the corrected threshold cycle of the bead-based approach in order to offset the influence of beads on the reaction. The corrected threshold cycle can be evaluated by *Ct*_s_ = *Ct*_b_ × log(1 + *E*_b_)/log(1 + *E*_s_), where *Ct*_b_, *Ct*_s_, *E*_s_, and *E*_b_ denote threshold cycle of the bead-based positive controls, threshold cycle of the corrected positive controls, solution-based PCR efficiency, and bead-based PCR efficiency, respectively.

### Characterization of the releasing efficiency of cDNA from the beads via on-chip photocleavage

**Figure 3:**
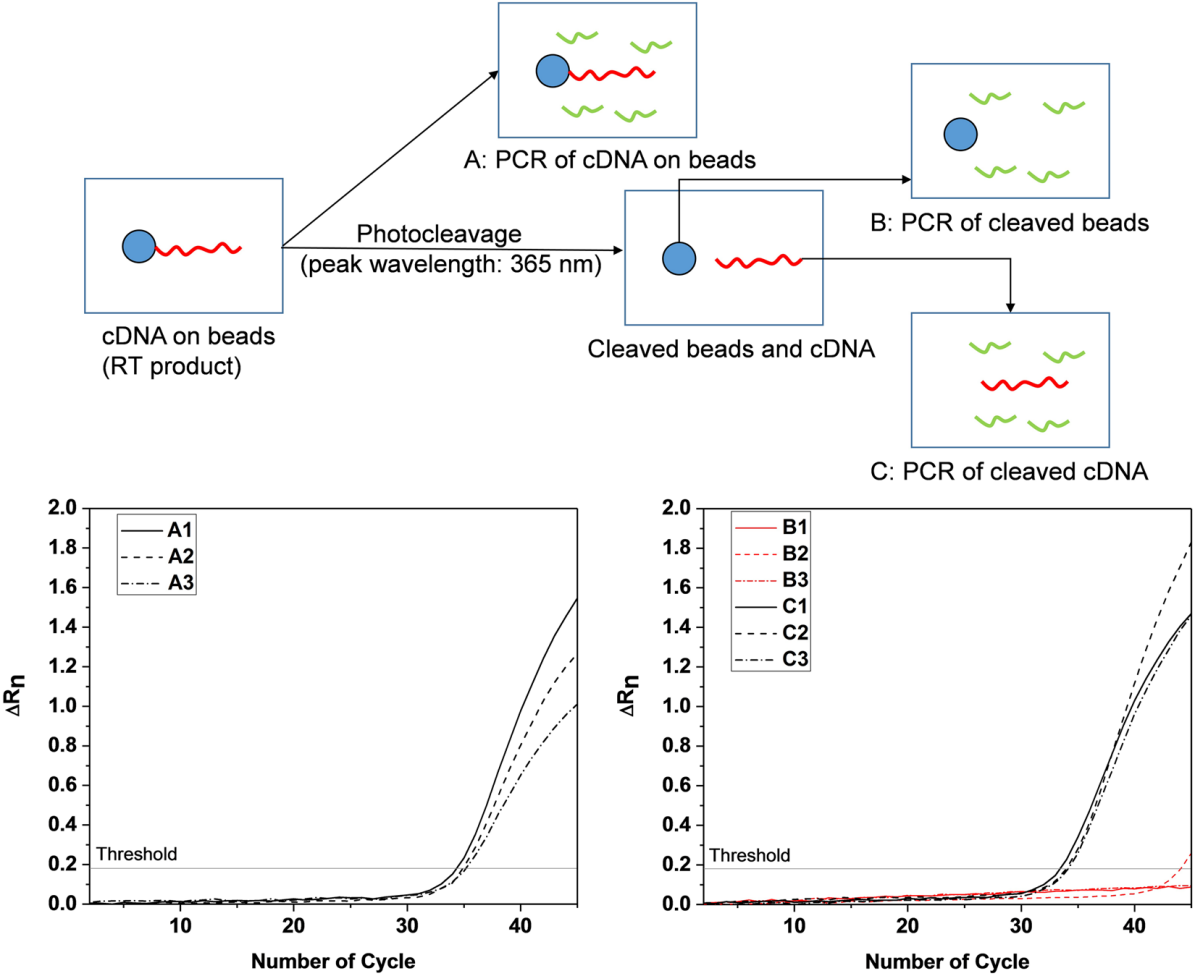
Schematic of experiment flow and qPCR following on-chip cDNA synthesis using magnetic beads, A: perform PCR with cDNA on beads, B: photocleave beads and run PCR with beads only, C: use the photocleaved cDNA product from B to run PCR. Solid, dashed and dash-dot curves represent independent experiments per experiment group.

It is also important to know the releasing efficiency of cDNA from the beads via on-chip photocleavage. Therefore, an experiment was designed to characterize the release efficiency (Fig. 3). The first arm of the experiment used on-chip cell lysis, mRNA capture, and RT processing of a small number (10) of IMR-90 cells obtained by dilution. Without photocleavage, the RT products (cDNA on beads) were collected and analyzed by qPCR. We next performed on-chip lysis of 10 cells, mRNA capture and RT, followed by on-chip photocleavage via UV irradiation (peak wavelength: 365 nm). The beads were separated from the cleaved RT product (cDNA), and qPCR analyses were performed separately using the cDNA cleaved from the beads and the remaining beads. Looking at the amplification curves for the housekeeping gene GAPDH, there is negligible amplification of the cleaved beads, indicating the cDNA has been effectively removed from the beads by the UV irradiation. We have also noticed that the threshold cycle *Ct*_b_ of cDNA-on-beads amplification (Positive Control: A1, A2 and A3) are higher than the *Ct* of cDNA in solution (C1, C2 and C3), which would be expected, given that we have already shown that the bead-based approach has a lower PCR efficiency than solution-based reactions (Fig. 3b). Evaluating the releasing efficiency of cDNA from the beads requires the corrected threshold cycle *Ct*_s_, which has been defined as previously defined. Thus, the releasing efficiency defined as (1 + *E*_s_)^−*Ct*^/(1 + *E*_s_)^−*Ct*s^ is estimated to be (mean ± s.d.) 95.6% ± 3.5% using experimentally determined cycle threshold (*Ct*_b_ and *Ct*) and PCR efficiency (*E*_s_ and *E*_b_).

### Microbeam-irradiated and bystander single cells

**Figure 4:**
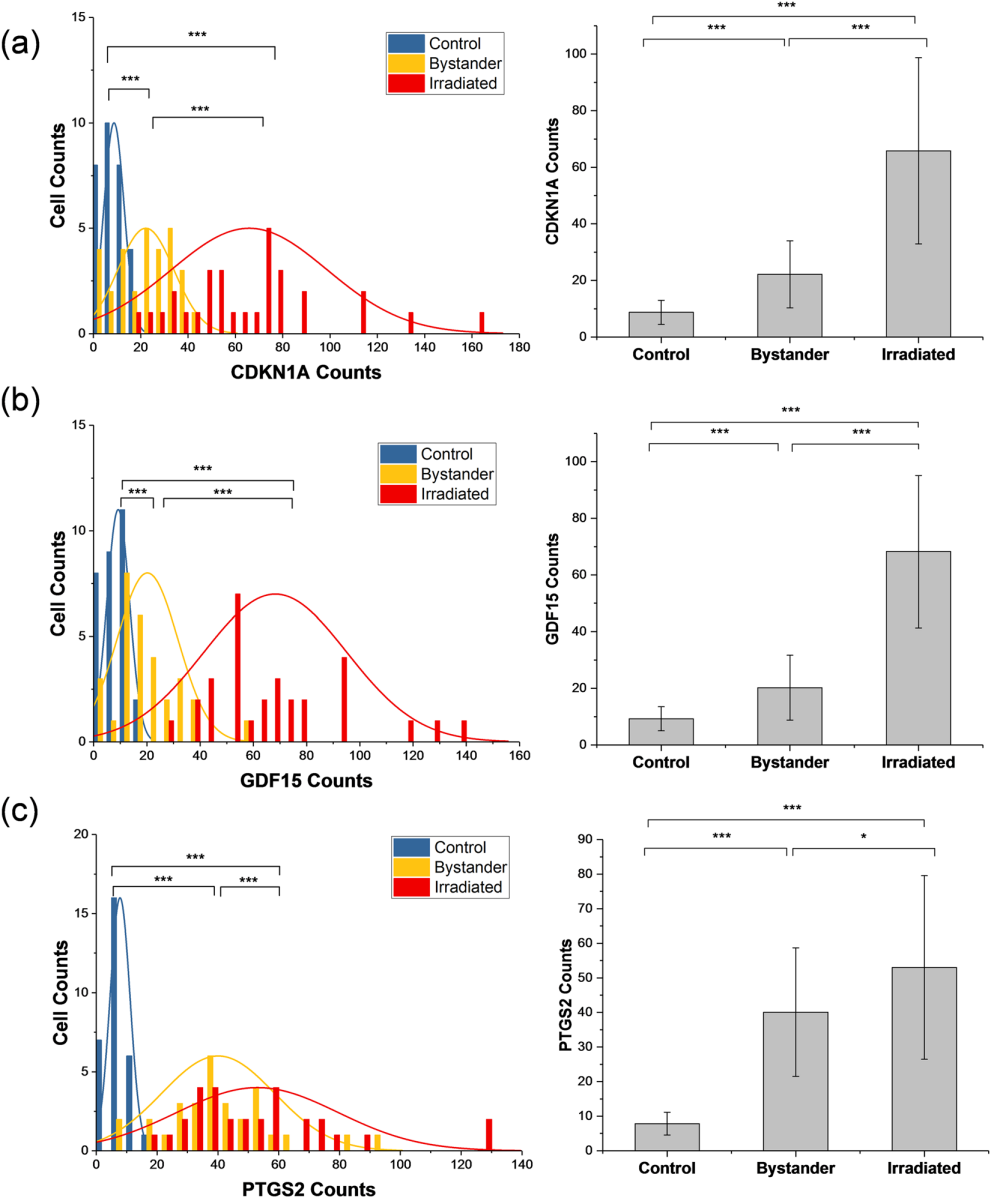
Left column: Significantly different distributions of transcript numbers of *CDKN1A*, *PTGS2* and *GDF15* as measured by digital PCR from individual control, bystander and microbeam-irradiated IMR90 cells. Right column: Comparisons of mean quantities of three gene products: *CDKN1A*, *PTGS2* and *GDF15* averaged over 30 individual control, bystander and microbeam-irradiated IMR90 cells. One asterisk (*) indicates p < 0.05 and three asterisks (***) indicate p < 0.001.

Having characterized the bead-based process on chip, we were ready to integrate the PreGEM chip with the cell picker and study the single-cell response to microbeam irradiation as well as the bystander effect. 30 Individual control, 30 microbeam-irradiated and 30 bystander single cells were one by one picked from microbeam dishes via the cell picker and transferred to the microchips and processed for cell lysis, mRNA capture, RT and photocleavage. The recovered cDNAs from the on-chip reaction were then used to run digital PCR reactions with analysis on the QuantStudio platform. The QuantStudio uses limiting dilution of the reaction mix to count individual molecules, providing absolute quantification of gene expression levels using Poisson statistics. Expression levels of radiation responsive genes *CDKN1A*, *GDF15* and *PTGS2* were measured to demonstrate the utility of our single-cell preprocessing pipeline in radiation studies. *CDKN1A* and *GDF15* are representatives of p53-regulated radiation response genes previously shown to respond predominantly in directly irradiated cells and not in bystanders^39,41^, while *PTGS2* as one of the most broadly studied genes in bystander studies^42–45^ is a representative of NFκB-regulated radiation response genes, and has been shown to respond almost identically in directly irradiated and bystander cells^39,41^. Figure 4 presents the distribution of individual control cells, bystander cells and irradiated cells based on the quantities of *PTGS2*, *CDKN1A* and *GDF15* in each cell. One interesting observation is the elevated variability in expression levels in both bystander cells and irradiated cells compared to controls. For example, the expression levels of *CDKN1A* among irradiated cells distribute in a range of 15 to 165 counts while bystander cells have a narrower range of 0 to 45 counts, compared to that the control cells fall within a range of 0 to 25 counts. Also, it was found that the expression levels of both *CDKN1A* and *GDF15* in irradiated cells are significantly higher than the expression levels for both genes in bystander cells (p < 0.001). The average fold-changes for *CDKN1A* and *GDF15* in irradiated cells are 7.56 and 7.37, respectively, while the average fold-changes for these two genes in bystander cells are 2.54 and 2.19, with respect to the mean value of the two genes in control cells. While for *PTGS2*, the average fold-changes are 6.77 and 5.24 in irradiated cells and bystander cells, respectively.

**Figure 5:**
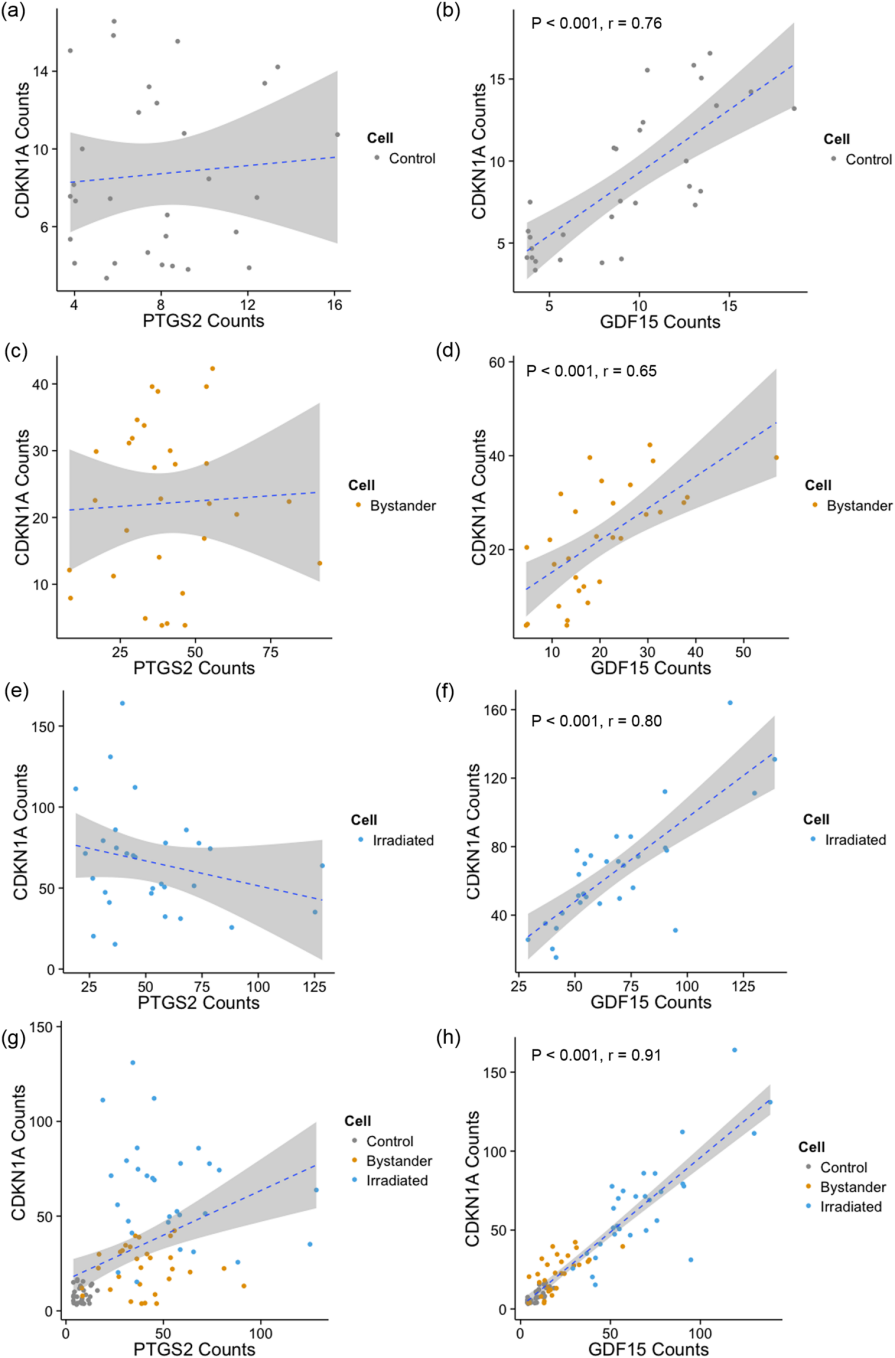
Scatter plots of expression levels of *CDKN1A* against *PTGS2* (**a**,**c**,**e**,**g**) and *CDKN1A* against *GDF15* (**b,d,f,h**). Grey shading represent 95% confidence level interval for predictions from a linear model (blue dashed line).

In order to compare the expression levels of the three genes within individual cells, regression analyses were applied to data points corresponding to expression levels of *CDKN1A* compared with those of *PTGS2* and *GDF15*. We found that a linear model was not significant to explain the relationship between the expression levels of *CDKN1A* and *PTGS2*, which represent two different signaling pathways, in individual control, bystander and irradiated cells (Fig. 5a,c,e). Conversely, expression of the two p53-regulated genes, *GDF15* and *CDKN1A*, was significantly correlated within individual cells (p < 0.05). Additionally, the *r* values were calculated in terms of the expression levels of *CDKN1A* and *GDF15* for sets of control, bystander and irradiated cells. A strong linear relationship was found for *CDKN1A* against *GDF15* among irradiated cells (*r* = 0.80), while the correlation decreased between these genes in bystander cells due to elevated variance (*r* = 0.65).

## Discussion

To support our microbeam studies of the radiation bystander effect, we have developed a system to perform single-cell gene expression measurements following irradiation with the microbeam. This system empowers our single-cell irradiation studies, allowing individual microbeam-irradiated cells or non-hit bystander cells to be tracked, lysed, and prepared for analysis. Our approach could also be applied to studies of rare cells in many other contexts. Gene expression variability in rare cells is of great interest for applications including cancer diagnosis^46^, cancer drug development^47^ and prenatal diagnosis^48^. While much can be learned through studying gene expression response in bulk cell populations, such analysis of gene expression dilutes the contribution of these rare cells to the pattern of the population. Information that covers the diversity and complexity of the cells as well as their unique molecular signatures is thereby lost.

The utility of the integrated preprocessing system was demonstrated by interfacing with QuantStudio digital PCR. The end-to-end analyses from microbeam irradiation to digital PCR gene expression measurements indicate cell-to-cell variability among individual control, microbeam-irradiated as well as bystander cells. It is critical to know that such variability is mainly due to the cell-to-cell differences in gene expression rather than being introduced by measurement noise. As can be seen from Fig. 4, the mean quantities of each gene in irradiated and bystander cells are greater than the mean of the gene in individual control cells. While the measurement noise would be expected to decrease with elevated abundance of transcripts, the variance in irradiated and bystander cells is larger than the variance in controls. This would indicate that there is minimal effect on dispersion from measurements and the variance of the single-cell data reflects actual cell-to-cell differences.

The response of lung fibroblast cells to radiation has been extensively studied by microarray and qRT-PCR^11,49–51^. For example, global gene expression four hours after bystander and direct alpha particle exposure of lung fibroblast cells was measured, revealing disparate roles of two major radiation response pathways, p53 and NFκB, in the bystander response. Activation of the p53 response pathway was found to be minimal in bystander cells, in contrast with the NFκB pathway, which appeared to respond identically in bystander and irradiated cells^39^. Additionally, lung fibroblasts were exposed to alpha-particle radiation at a dose range of 0–1.5 Gy, after which the *GDF15* gene, regarded as a marker for lung injury, was assessed at the protein level and 3-fold higher expression levels were found in exposed cell culture media 24 hours after exposure^52^. The single-cell data presented in Fig. 4 are consistent with these studies, which could be explained by the previous observation that p53-regulated genes like *CDKN1A* and *GDF15* were expressed at elevated levels in directly irradiated cultures, while showing little or no change in bystanders^39^. In contrast, the response pattern of NFκB regulated genes such as *PTGS2* was found to be virtually identical in bystander and irradiated cells.

What was missed in the above studies was the heterogeneity of responses within bystander and irradiated populations. While studies in populations gave an averaged cellular response, it is clear that the presented genes were not uniformly expressed across even a small population of cells (*n* = 30) within each treatment set. Within irradiated cells, expression levels for any particular gene may not be uniformly elevated among the cells, in addition to inherent cell-to-cell differences revealed in the controls (Fig. 4). Signaling and response mechanisms in bystander cells are complex, involving both direct contact and gap junctions between bystanders and irradiated cells^53,54^ as well as communication through extra-cellular signals in the culture media^55,56^. Thus, it is possible that the distance between an irradiated cell and a non-hit bystander, the concentration gradient of signaling molecules in the media, and the movement of the media could contribute to the observed differences in gene expression across bystander cells.

Within the same signaling pathway, *CDKN1A* and *GDF15* both are p53-induced genes^57^, and it is clear from Fig. 5h that they are significantly correlated in microbeam-irradiated cells, and to a lesser extent in control and bystander cells. It is known that *GDF15*, which contains two p53 binding sites in its promoter region, is a direct target gene of p53^58^. Radiation-induced DNA damage can induce *GDF15* expression in a p53-dependent manner. It has also been found that *GDF15* holds a moderate but significant association with p53 after DNA damage^59^, which would indirectly reveal the correlation of genes *CDKN1A* and *GDF15* within the p53 pathway.

Next consider the pair of *CDKN1A* and *PTGS2*, which represents activation of different pathways including p53 and NFκB. The former gene plays essential roles in the DNA damage response^60^ and the latter counteracts p53 activity as well as inhibits DNA damage-induced apoptosis^61,62^. As seen from Fig. 5, the correlations between the two genes in controls and bystanders are weak and positive while the two genes were found to be negatively related in irradiated cells. The negative trend observed between *CDKN1A* and *PTGS2* in irradiated cells may imply a transcriptional cross talk between NF-κB and p53, in which both pathways inhibit each other’s ability to stimulate gene expression and this process is likely dependent on the relative levels of the two transcripts^63^. It has been suggested that stimulation of NF-κB promotes resistance to programmed cell death while the activation of p53 is associated with the induction of apoptosis or cell cycle arrest following DNA damage^64,65^. If this is indeed the case, it is possible that NF-κB activation occurs simultaneously with the induction of p53 in a few individual cells shown in Fig. 5e, which exhibited equally high levels of expression of both genes, but that activation of the two pathways is not tightly linked within the same cell.

## Conclusions

The data presented here demonstrate the integrated preprocessing approach for single-cell irradiation studies, coupled to qPCR or digital PCR, provides a powerful method to investigate the variability of gene expressions in rare cells with microbeam irradiation, and many other applications where rare cells are of interest.

## Methods

### Cells

Normal human diploid lung fibroblasts (IMR-90) were obtained from the American Type Culture Collection (ATCC® CCL-186; Manassas, VA, USA). Cells at passage 10–12 were grown in Eagle’s Minimum Essential Medium (CellGro, Manassas, VA, USA) supplemented with 12.5% heat-inactivated (56 °C, 30 min) fetal calf serum, 400 mM L-alanyl-L-glutamine, 100 U/ml penicillin and 100 µg/ml streptomycin (Sigma-Aldrich Corp., St. Louis, MO, USA). The cells were maintained at 37 °C in a humidified incubator with 5% CO_2_ in air.

### Cell irradiation

**Figure 6:**
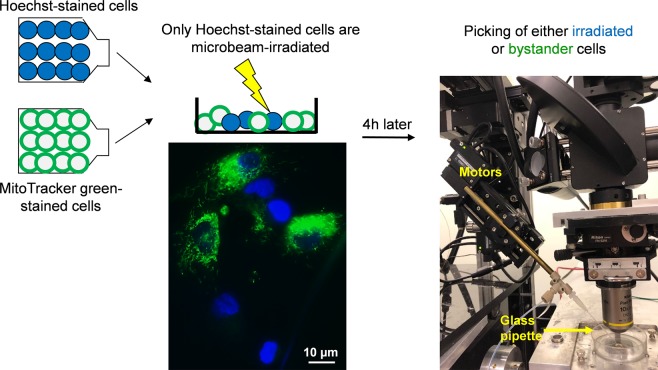
A co-culture of Hoechst-stained IMR-90 cells (blue) with MitoTracker green-stained IMR-90 cells (green) was established on microbeam dishes; at the endstation only Hoechst-stained cells were irradiated while the cytoplasmic-stained cells were designated as bystanders; single-cell picking was conducted 4 h after irradiation using a capillary micropipette with computer controlled positioning motors.

Microbeam irradiations were performed at the Radiological Research Accelerator Facility (RARAF)^24^, Columbia University. Twenty-four hours before the experiment, cells were seeded in two 12.5 cm^2^ flasks at 70–80% confluence. The day of the experiment, one flask was incubated for 30 minutes with 100 nM Hoechst 33342 and the other with 200 nM MitoTracker Green (Thermo Fisher Waltham, MA USA). Cells from both flasks were then trypsinized and plated 1:1 on custom-made microbeam dishes^20^ and allowed to attach for at least 1 h (Fig. 6). Immediately before irradiation, the culture medium was removed from the microbeam dish. The microbeam dish was positioned adjacent to the microbeam exit window and a moisture containment collar was placed over the objective lens to keep the cells from becoming dehydrated during the approximately 15 min irradiation time. The RARAF microbeam system has integrated image analysis, which was used to visualize the nuclei stained with Hoechst 33342. A computer automated nuclear irradiation protocol found the location of every stained nucleus on the dish. We used the RARAF 5 MV Singleton Accelerator to deliver five doubly charged helium particles (He^2+^), simulating an alpha particle, with an energy of 6.0 MeV to the center of each stained nucleus. The ion beam was measured to have a width of <5 µm for all irradiations, and based on SRIM calculations (Stopping Range of Ions in Matter)^66^, each He^2+^ particle had a linear energy transfer (LET) of approximately 100 keV/µm. Cells showing nuclear stain were microbeam-irradiated while the MitoTracker Green-stained cells were not targeted and therefore were designated as “bystander” (Fig. 6). The particle fluence was measured by a gas proportional counter positioned above the cells. After every nuclear stained cell on the plate had been irradiated the dish was removed from the irradiation endstation, fresh medium was added to the dish, and the dish was placed in the incubator at 37 °C until ready for cell picking at 4 hours post irradiation. All control cells were stained with Hoechst 33342 and sham irradiated.

### Cell picking

Cells were viewed on a custom microscope using a 10x objective with live imaging (pco.edge 5.5, PCO, Kelheim, Lower Bavaria, Germany). Custom software allowed for imaging and lighting control as well as computer adjustment of the position of the pipette mounted on a 6 axis stage (X, Y, Z, azimuth, elevation and retraction; Zaber Technologies Inc., Vancouver, BC). 40 uL of 0.25% trypsin-EDTA was added to the microbeam dish prior to picking to aid in cell release. To pick a cell, the pipette was positioned directly adjacent to a cell of interest on the microbeam dish. Multi-color illumination, with the use of a Lumencor Aura Light Engine (Lumencor, Beaverton, OR), a light source (Morrell Instruments, Mellville, NY) and a camera (PCO-Tech Inc, Romulus, MI), allowed for simultaneous imaging of both the nuclear stain and cytoplasmic stain, thus permitting determination of the cell as “irradiated” or “bystander”, respectively. Single cells were aspirated into the micropipette along with 1–3 µL of 0.25% trypsin-EDTA. After picking a cell, the micropipette was moved out of the microbeam dish and positioned within the input well of a PreGEM chip, where the picked cell was ejected directly into lysis buffer (Thermo Fisher, Waltham, MA).

### Fabrication of PreGEM chip

**Figure 7:**
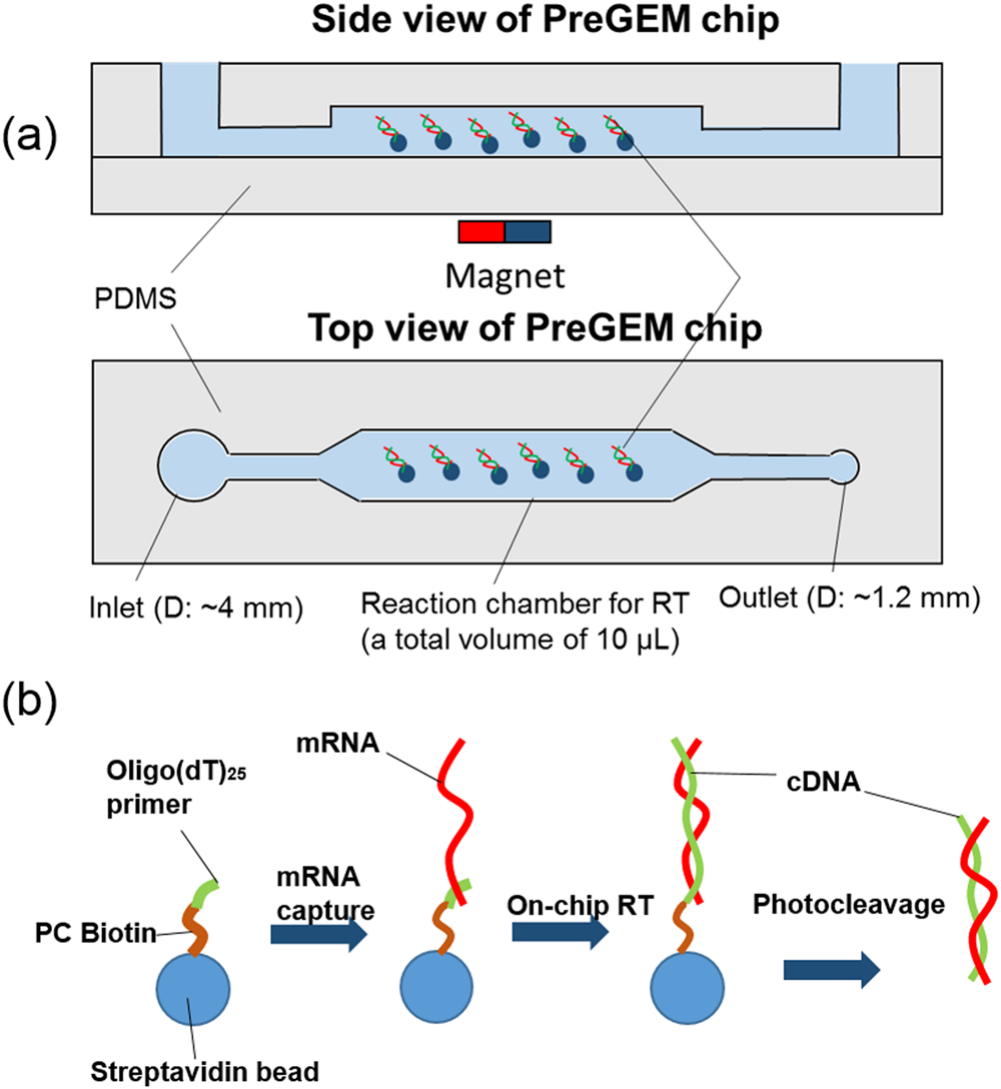
(**a**) Schematic of the side view of a PDMS PreGEM chip made by soft lithography. The microfluidic chip consists of an inlet for introduction of reagents, cell lysis and mRNA capture using magnetic beads as well as a reaction chamber for on-bead RT. (**b**) a bead-based process on the PreGEM chip: mRNA capture by Oligo(dT)_25_ on beads, cDNA synthesis on beads during RT, and cDNA release by photocleaving the PC (photocleavable) Biotin linker on beads.

The chip (Fig. 7a) is fabricated using standard soft lithography microfabrication techniques^67^. SU-8 photoresist (MicroChem Corp., Newton, MA) was spun-coated on a silicon wafer and patterned with photolithography to define the liquid channel mold. Then, PDMS (Dow Corning) was poured over the mold and an additional vapor barrier^33^ was embedded in the PDMS. Sheets bearing the microfluidic features were then peeled off the mold followed by inlet and outlet hole punching. The molded PDMS was sealed to a glass slide using oxygen plasma bonding.

### On-chip RNA capture

Different copy numbers of XenoRNA template (2 × 10^4^, 5 × 10^4^ and 10^5^, Thermo Fisher Scientific Inc.) were introduced to the microchip, and were captured by 3.5 × 10^6^ beads (Thermo Fisher Scientific Inc). Separating the magnetic beads from the remaining solution using a magnet, the effluent solution was transferred to micro tubes and mixed with another solution containing 3.5 × 10^6^ beads, which was followed by off-chip RT. In the end, 40-cycle qPCRs were run to detect the amount of un-captured templates.

### On-chip RT characterization

The efficiencies of the on-chip bead-based RT were investigated using various copy numbers of XenoRNA templates (2 × 10^4^, 5 × 10^4^ and 10^5^, Thermo Fisher Scientific Inc.). The on-chip RTs were first conducted after mixing RT reagents, XenoRNA, biotinylated oligo(dT)_25_ primer (Integrated DNA Technologies Inc.) and 3.5 × 10^6^ streptavidin magnetic beads. Subsequently, PCRs were performed with the products of RT using the qPCR system. In comparison for the on-chip efficiency, in-tube RTs were performed using a thermocycler followed by qPCR characterization. Additionally, to characterize the effects of beads on reaction efficiency, bead-based PCRs were subsequently performed with the products of in-tube RT by mixing and binding biotinylated cDNA to streptavidin beads, comparing the bead-based approach with the standard bench-top solution-based approach.

### Single-cell processing on the PreGEM chip

The input well of the PreGEM chip was pre-filled with the lysis buffer, so as to immediately lyse the input cell after it was picked and placed within the input well. Next, mRNA templates in the cell lysate were collected by photocleavable 5′-PC Biotin-(dT)_25_-3′ bead tether, which was made by incubating magnetic streptavidin beads (Thermo Fisher Scientific Inc.) with photocleavable biotinylated oligo(dT)_25_ (Integrated DNA Technologies Inc.). The principle of mRNA capture relies on base pairing between the polyA tails of the mRNA and oligo(dT)_25_ immobilized on the surface of the beads. In the reaction of reverse transcription (RT), the bead-bound oligo(dT)_25_ functions as a primer for synthesis of cDNA. Extracted mRNA were moved from inlet well to reaction chamber using a magnet, followed by the introduction of reverse transcription mix (dNTP, RT buffer, MgCl_2_ solution, Reverse Transcriptase, RNase inhibitor). After RT (10 min at 25 °C and 50 min at 42 °C), the microchip was exposed under a UVA lamp (Sylvania Inc) photocleaving the PC linker, which released the cDNA from the beads.

### In-tube RT, qPCR and digital PCR (dPCR)

In-tube RTs were run using a thermocycler (Eppendorf) with a standard protocol (10 min at 25 °C and 50 min at 42 °C). qPCRs were performed using 96-well reaction plates run on the 7900HT Fast Real-Time PCR (Applied Biosystems). 5 μL RT products, 6.25 μL nuclease-free water, 1.25 μL Taqman gene expression assay (Hs99999905_m1, Thermo Fisher Scientific Inc.) and 12.5 μL Taqman gene expression master mix were loaded on the plates, which were run for 45 cycles. The QuantStudio 3D digital PCR system (Thermo Fisher Scientific Inc.) was used to conduct digital PCRs. Starting samples in a final volume of 15 μL that consisted of bead-excluded RT product, Taqman gene expression master mix (Thermo Fisher Scientific Inc.), Taqman gene expression assay (Hs99999142_m1, Hs00153133_m1, Hs00171132_m1, Thermo Fisher Scientific Inc.) and nuclease-free water were loaded onto Digital PCR Chips (Applied Biosystems) via the QuantStudio 3D Digital Chip Loader (Applied Biosystems). The chips were immersed in oil to avoid evaporation and sealed with UV sealant (Applied Biosystems). This was followed by running 40 cycles of dPCR using GeneAmp® PCR system 9700 Thermocycler, which is adapted for digital PCR reactions. The chips were then loaded onto the QuantStudio™ 3D Digital PCR Instrument, and the end-point fluorescence of each well on the chips was measured using QuantStudio™ 3D Digital PCR software v3.0. The fluorescence data were read and analyzed using QuantStudio 3D Analysis Suite Cloud Software.

### Statistical analysis

Statistical differences between two groups of cells were determined using unpaired two-sample t-test or one-way ANOVA followed by post-hoc Tukey test, where necessary. Pearson correlation coefficient (*r* value) was used to measure the linear relationship between expression levels of two genes. Defined as the covariance of two variables divided by the product of their standard deviations and calculated as follows: $r=\frac{{\sum }_{i=1}^{n}({x}_{i}-\bar{x})({y}_{i}-\bar{y})}{\sqrt{{\sum }_{i=1}^{n}{({x}_{i}-\bar{x})}^{2}{\sum }_{i=1}^{n}{({y}_{i}-\bar{y})}^{2}}}$, where *x* and *y* represent variables and n is the number of experiments. The Kolmogorov-Smirnov test was used to test the normality of the samples and to decide whether the distribution functions of two samples are significantly different. R (version 3.1.1) was used to perform statistical analysis.
